# Supplementary material for: Hidden Rice Diversity in the Guianas
Source: Front Plant Sci. 2019 Sep 20;10:1161. doi: 10.3389/fpls.2019.01161 (PMC6764085; doi:10.3389/fpls.2019.01161)
Supplement: Supplementary file 1 [file DataSheet_1.docx]

Supplementary Material

# Supplementary Figures


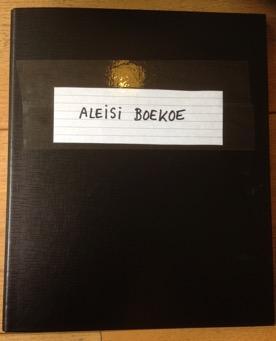
  
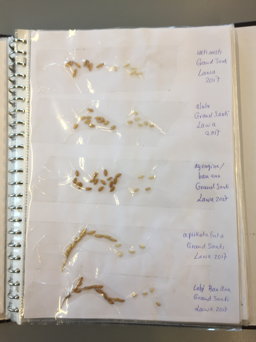


**Figure S1 | (A)** Ring binder named ‘aleisi boekoe’ (rice book in Sranantongo). **(B)** Pages containing different rice varieties. Picture by Tinde van Andel.


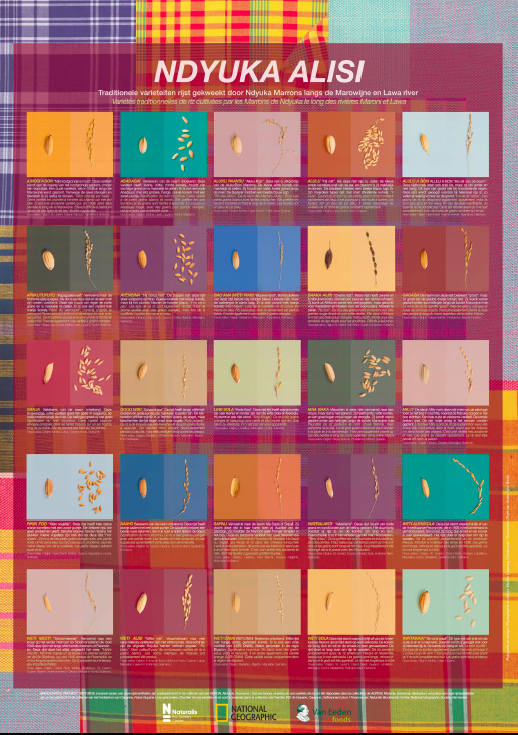


**Figure S2** **|** Poster with pictures of the different rice varieties and associated traditional information in Dutch and French to be distributed in Suriname and French Guiana. Photography and design by Marlies Lageweg.

**
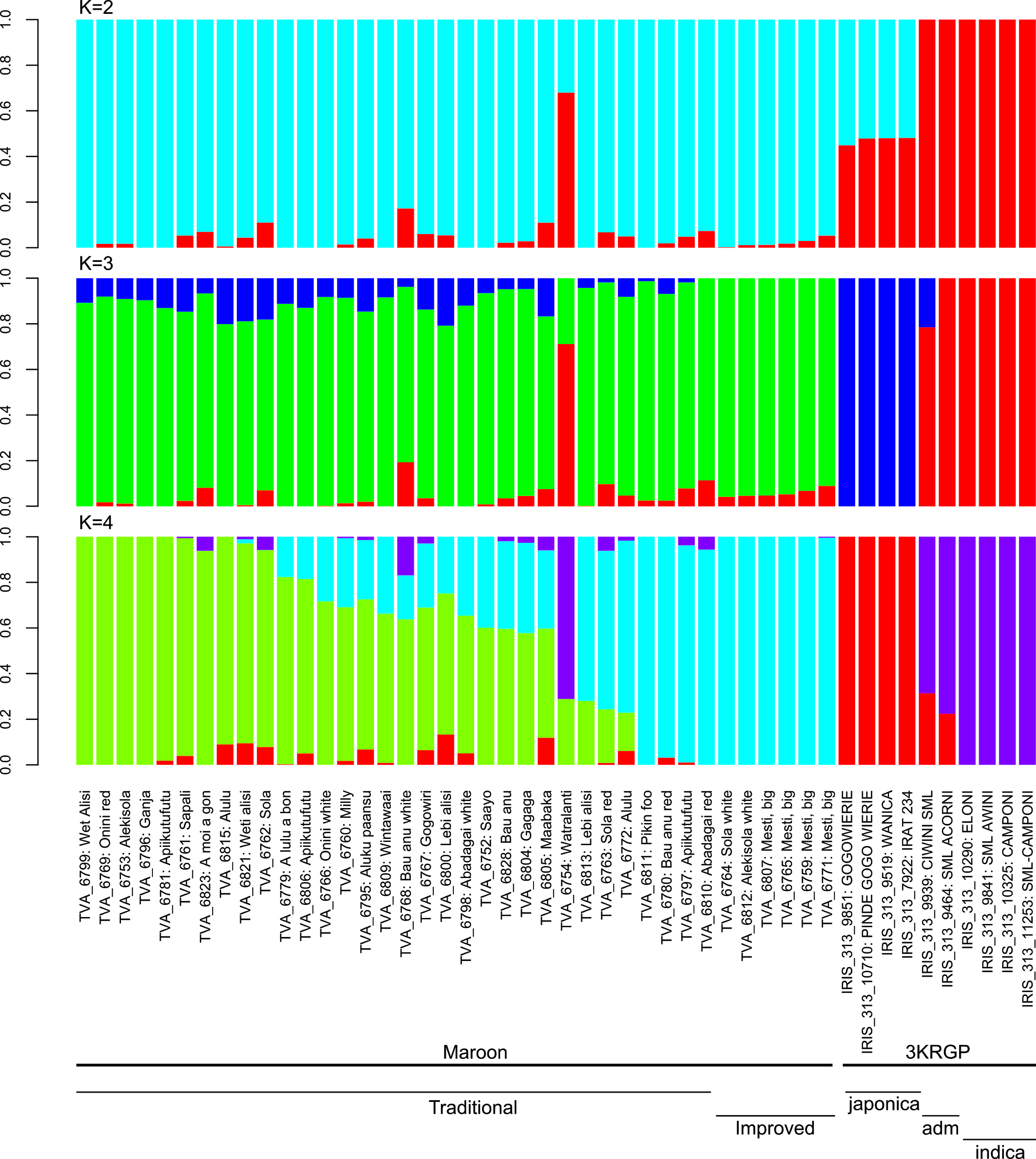
**

**Figure S3 |** Population structure results for all primary (Maroon) and secondary (3KRGP) *O. sativa* accessions, based on 64,313 SNPs. **(A)** Cross-validation error estimates demonstrate that the optimal number of ancestral populations given the data is K=2.


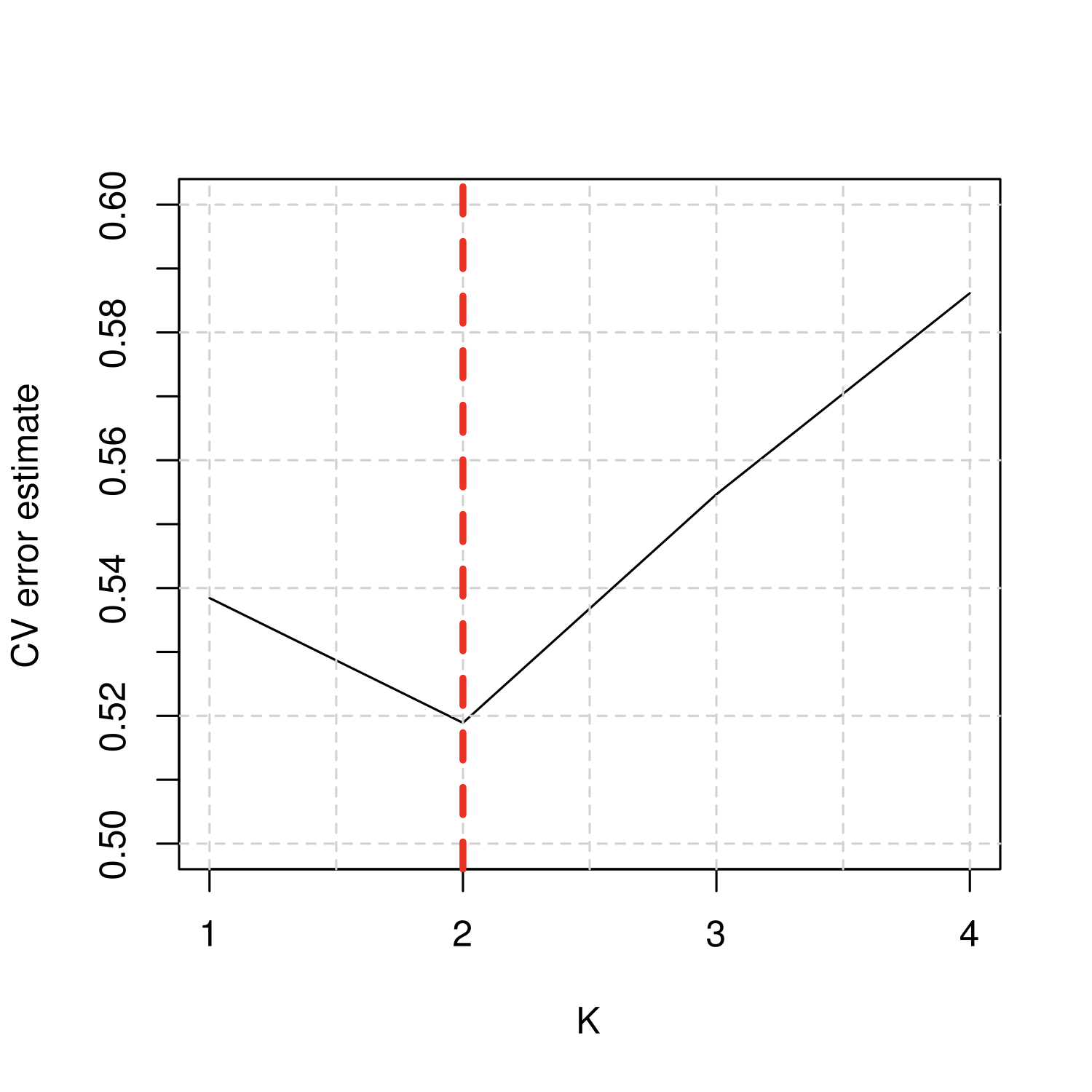


**(B)** Relative ancestry fractions per individual at different levels of K.


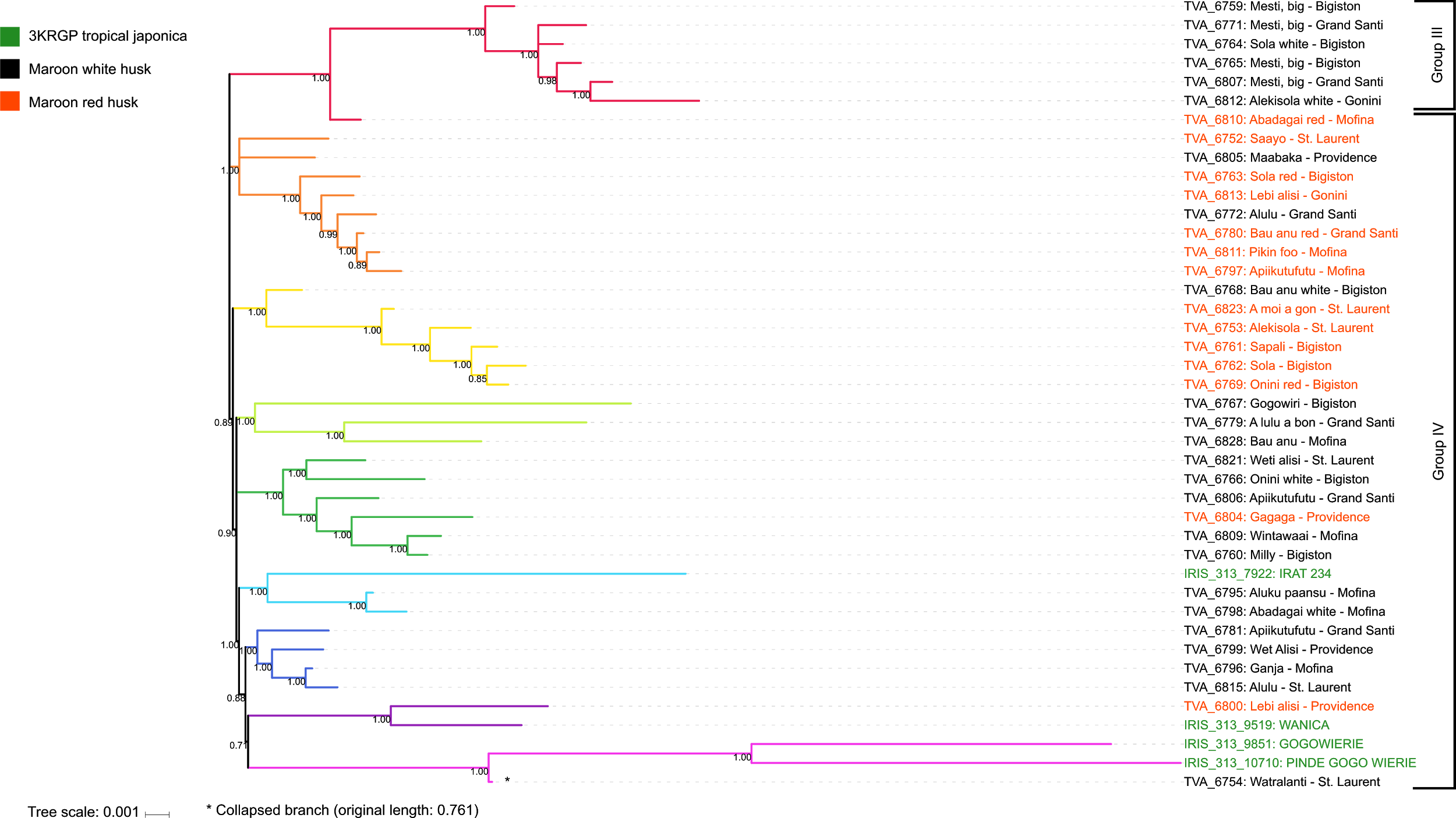


**Figure S4 |** Clustering of the Maroon Asian rice gene pool. **(A)** Approximately ML tree based on 39,595 homozygous SNPs, with branch lengths representing evolutionary distances between all Maroon and 3KRGP tropical japonica varieties. The branch connecting to the sole wetland variety is collapsed for visualization purposes and marked with an asterisk. Clades with good support values (>0.85) on all internal nodes have been identified and labelled with different colors.


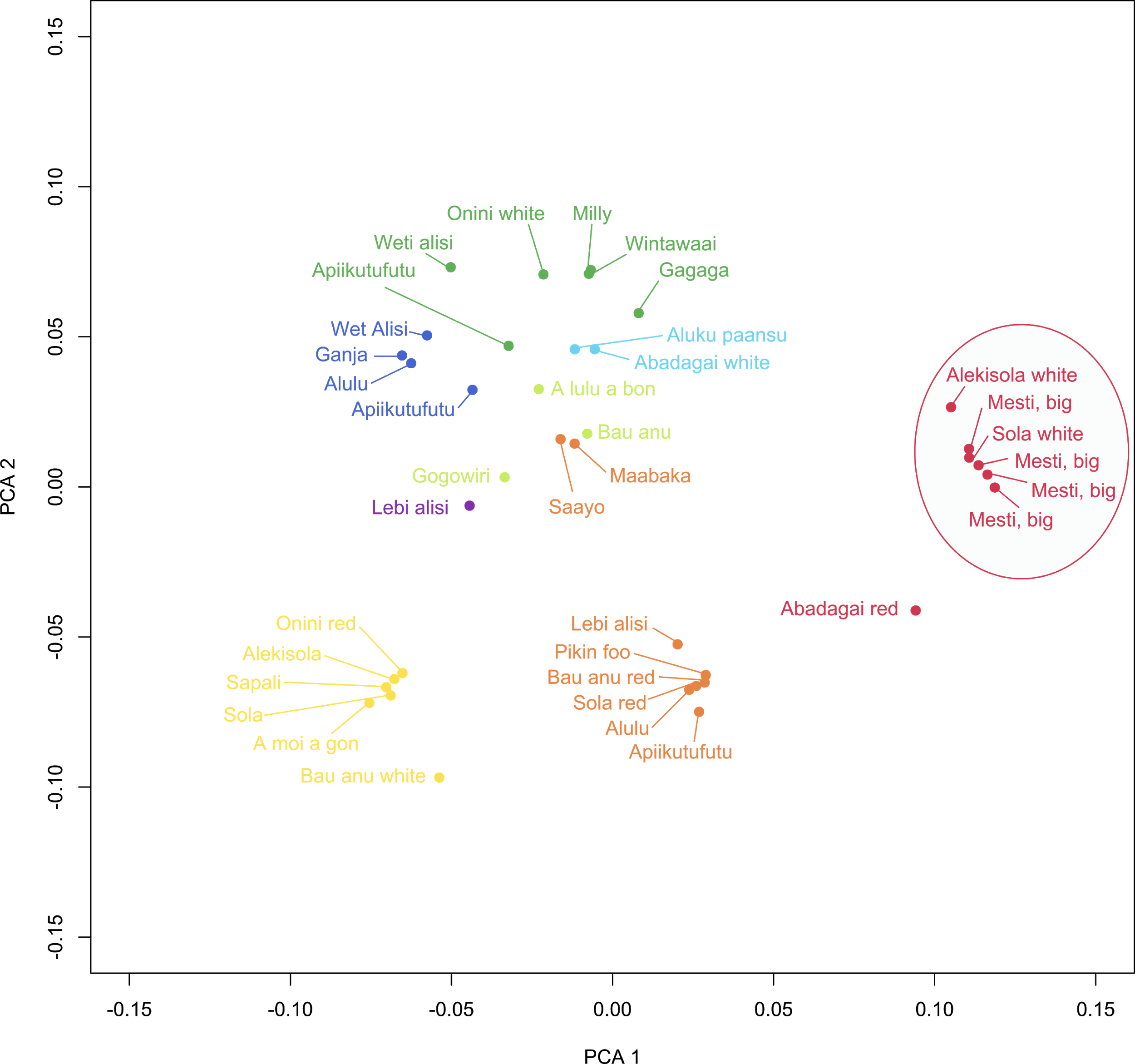


**(B)** Multi-dimensional scaling in two dimensions of raw Hamming distances for all improved (Group III) and traditional dryland (Group IV) varieties. Individuals are labelled by vernacular name and color-coded based on the clustering in **Figure S4A.** Improved varieties are circled.
