# Supplementary material for: Hidden Rice Diversity in the Guianas
Source: Front Plant Sci. 2019 Sep 20;10:1161. doi: 10.3389/fpls.2019.01161 (PMC6764085; doi:10.3389/fpls.2019.01161)
Supplement: Supplementary file 2 [file Table_1.docx]

**Supplementary Table S1.** Field questionnaire, based on the 'rice passport data sheet' developed by the SNRI-ADRON, Suriname**.**

| **Field** | **Units / levels / comments** | **Translation Aucan / Sranantongo** |
| --- | --- | --- |
| Collecting organizations | Naturalis Biodiversity Center | (only when relevant) |
| Collectors' names | Tinde van Andel; Alice Bertin |  |
| Collection number | TvA + unique number |  |
| Collecting date |  |  |
| Farmer's name | Local script + English script if different |  |
| Language |  |  |
| Ethnic group |  |  |
| Province | or equivalent top-level sub-national division |  |
| District | or equivalent second-level sub-national division |  |
| Nearest town |  |  |
| Locality description | e.g. 10 km S along road from A to B |  |
| Location used for GPS measurement | 1=collecting site; 2=nearby |  |
| Datum set on GPS | preferably WGS84 (used by Google Earth) |  |
| Latitude | dd mm.mmm h |  |
| Longitude | ddd mm.mmm h |  |
| Altitude | m above sea level |  |
| Source | 1=field; 2=threshing floor ; 3=farm store ; 4=market place |  |
| Variety name | In local script, and also transliterated into English script if different | San a nen fu disi alisi ya? |
| Meaning of variety name | literal translation | San beteken disi nen? San wani taki dati? |
| Interpretation of variety name | The sense intended to be conveyed by the name | San ede yu kai gi en a nen ya? |
| Species* | check ligule! Elongated is O. sativa, short is O. glaberrima |  |
| Variety group of sativa | 1=indica; 2=japonica |  |
| General topography | 1=swamp; 2=flood plain; 3=Undulating; 4=Hilly; 5=Mountainous |  |
| Local topography | 1=Level; 2=Slope; 3=Summit; 4=Depression; 5=Valley |  |
| Site levelling | 1=Terraced; 2=not terraced |  |
| Toposequence | 1=lower; 2=middle; 3=upper |  |
| Soil texture | 1=Sandy; 2=Loamy; 3=Clayey; 4=Silty; 5=Organic |  |
| Soil fertility | 1=fertile; 2=moderate; 3 poor |  |
| Sampling method | 1=Random; 2=non-Random |  |
| Varietal sample | 1=single variety; N=mixture of N varieties |  |
| Abundance | 1=Abundant; 2=Frequent; 3=Occasional; 4=Rare |  |
| Origin | 1=Local; 2=introduced |  |
| Introduced origin | If origin is introduced, state where introduced from | Pe a alisi ya komoto? Pe yu fende a alisi ya? |
| Source of seed | From whom / where did the farmer get the seed? 1=own harvest; 2=market place; 3=friend or relative | Pe yu fendi a sii / paansu? A yu eigi gron? Yu bai en a wowoyo? Yu famiri / mai gi yu? |
| Acquisition of seed | How did the farmer get the seed? 1=saved; 2=bought; 3=bartered/exchanged; 4=received as gift | Yu kengi (a sii) anga sama? |
| Sowing date |  | Onten yu be paandi a alisi ya? |
| Maturity date |  | Onten a alisi ya bun fu koti? Baka onmeni mun a lepi? |
| Harvest date |  | Onten yu e go koti a alisi ya? |
| Method of harvest | 1=cut stem; 2=cut panicles; 3=strip grain; 4=other |  |
| Usage | 1=Main rice dish; 2=Noodles; 4=Snacks; 8=Sweet rice; 16=Alcohol; 32=Cultural ceremonies; 64=other (medicine, to sell, specify). | Yu e nyan a alisi ya? Yu ka seli a alisi ya? A fu gi yorka nyanyan? / gadu nyanyan? Nu fa meki dresi tu? |
| Cooking quality | 1=Excellent; 2=Good; 3=moderate; 4=bad | A makiliki fu boli? A teki langa fu boli? |
| Eating quality | 1=Excellent; 2=Good; 3=moderate; 4=bad | A bun fu nyan? |
| Farmer's description of variety | What are the special attributes of this variety? | Sortuwan sani de speciaal fu disi aleisi? |
| Best characteristics of this variety* |  | San na a wini pisi fu disi aleisi? |
| Worst characteristics of this variety* |  | Sortuwan sani de probleem fu disi aleisi? |
| Milling quality | milling by hand: 1=easy, 2=average,3= difficult | A alisi ya makilili fu mii / fon? |
| Aroma | 1=aromatic; 2=non-aromatic | A e sumee switi? |
| Tolerance to abiotic stress | 1=drought; 2=floods; 4=salinity; 8=others | A alisi lobi dee ten efu alen ten? |
| Other abiotic stresses | If score above = 8, list other abiotic stresses to which it is tolerant |  |
| Tolerance to | 1=weeds; 2=insects; 4=diseases; 8=others (birds?) | Foo lobi a alisi fy nyan? A alisi ya kisi wan siki? |
| Other biotic stresses | If score above = 8, list other biotic stresses to which it is tolerant |  |
| Cultural type | 1=irrigated; 2=rainfed lowland; 3=upland; 4=deep water; 5=swamp |  |
| Cultural practice | 1=shifting; 2=terraced; 3=direct seeding; 4=double transplanting; 5=mixed |  |
| Farmer's varieties | List other varieties used by the farmer |  |
| Farmer's sequence of sowing & harvesting | Which variety is sown first, which one is ripe first? | Sortuwan alisi you prani fosi? Sortuwan alisi de lepi fosi? |
| Use of non-food rice types* | Do you know rice types grown only for the birds or ancestors? | Sortuwan alisi yu prani soso fu den foo / yorka? |
| Farmer's environments | Describe the environments used by the farmer |  |
| Does farmer know early shattering rice?* | Describe varieties that drop their seeds so that making bundles is difficult | Sortuwan alisi abi sii di fadon snel? Dat yu no man meki bosu? |
| Farmer's matching of variety to micro-environment | Does the farmer have a specific reason for choosing this variety for this field? What? | Fu san ede yu prani disi alisi ini disi presi? |
| Farmer's matching of management practice to specific environment | Does the farmer have a specific reason for this management for this field? What? |  |
| Seed selection practice | If the farmer is using farm-saved seed, describe the farmer's practice for selecting seed from one crop for the next season's crop | Sortuwan siri yu teki fu paansu? |
| Seed storage practice | Describe how the farmer stores seed | Pe yu kibii yu paansu? |
| Gender issues | Describe what farming work is done by the men/ women | Sortuwan alisi wooko man du / uman du? |

* Additional questions to the standard SNRI/ADRON questionnaire
